# Adverse Obstetric and Perinatal Outcomes following Treatment of Adolescent and Young Adult Cancer: A Population-Based Cohort Study

**DOI:** 10.1371/journal.pone.0113292

**Published:** 2014-12-08

**Authors:** Fatima A. Haggar, Gavin Pereira, David Preen, C. D'Arcy Holman, Kristjana Einarsdottir

**Affiliations:** 1 The Department of Surgery, The Ottawa Hospital Research Institute, The University of Ottawa, Ottawa, Canada; 2 Centre for Health Services Research, School of Population Health, The University of Western Australia, Perth, Australia; 3 Telethon Kids Institute, The University of Western Australia, Subiaco, Australia; 4 Center for Perinatal Pediatric and Environmental Epidemiology, School of Medicine, Yale University, New Haven, Connecticut, United States of America; Center for Human Reproduction, United States of America

## Abstract

**Objective:**

To investigate obstetric and perinatal outcomes among female survivors of adolescent and young adult (AYA) cancers and their offspring.

**Methods:**

Using multivariate analysis of statewide linked data, outcomes of all first completed pregnancies (n = 1894) in female survivors of AYA cancer diagnosed in Western Australia during the period 1982–2007 were compared with those among females with no cancer history. Comparison pregnancies were matched by maternal age-group, parity and year of delivery.

**Results:**

Compared with the non-cancer group, female survivors of AYA cancer had an increased risk of threatened abortion (adjusted relative risk 2.09, 95% confidence interval 1.51–2.74), gestational diabetes (2.65, 2.08–3.57), pre-eclampsia (1.32, 1.04–1.87), post-partum hemorrhage (2.83, 1.92–4.67), cesarean delivery (2.62, 2.22–3.04), and maternal postpartum hospitalization>5 days (3.01, 1.72–5.58), but no excess risk of threatened preterm delivery, antepartum hemorrhage, premature rupture of membranes, failure of labor to progress or retained placenta. Their offspring had an increased risk of premature birth (<37 weeks: 1.68, 1.21–2.08), low birth weight (<2500 g: 1.51, 1.23–2.12), fetal growth restriction (3.27, 2.45–4.56), and neonatal distress indicated by low Apgar score (<7) at 1 minute (2.83, 2.28–3.56), need for resuscitation (1.66, 1.27–2.19) or special care nursery admission (1.44, 1.13–1.78). Congenital abnormalities and perinatal deaths (intrauterine or ≤7 days of birth) were not increased among offspring of survivors.

**Conclusion:**

Female survivors of AYA cancer have moderate excess risks of adverse obstetric and perinatal outcomes arising from subsequent pregnancies that may require additional surveillance or intervention.

## Background

In general, cancers that commonly occur among adolescents and young adults (AYAs), ages 15–39 years, have a relatively good prognosis [1]–[3]. For several of these malignancies (e.g., melanoma, Hodgkin's lymphoma, thyroid and testicular cancers), 5-year relative survival is in excess of 85% [1]. Recent improvements in therapy and early detection of other common AYA malignancies, such as carcinomas of the breast and cervix, have the potential to further increase overall cancer survival in this age group [4], [5]. These advances will inevitably lead to increases in the number of cancer survivors, who will potentially be faced with late and long-term physical morbidity, as well as psychological and psychosocial challenges [4], [6]. Female survivors considering pregnancy are faced with further concerns about the impact of cancer therapy on their ability to maintain normal pregnancy and the possibility of adverse outcomes among their offspring [7]. These effects may be manifested as an increase in obstetric complications or an increase in the frequency of adverse neonatal outcomes, such as low birth weight, small for gestational age and congenital malformations. In light of the recent trend of delayed childbearing for personal, educational or professional reasons [8], [9], evaluating the risks of pregnancy outcomes following treatment for cancer is of increasing importance. Several institutions have reported their experience with long-term survivors of diverse types of pediatric cancer [10]–[20]. However, there is a paucity of studies focusing specifically on patients of childbearing age at the time of cancer diagnosis. This population-based study investigated the occurrence of selected adverse pregnancy and neonatal outcomes respectively, among females diagnosed with cancer when aged 15–39 years and their offspring.

## Methods

### Health datasets

The Western Australian Data Linkage System (WADLS) was used to extract health records of all females diagnosed with cancer in Western Australia (WA) during the period 1st January 1982 to 31st December 2007, as well as a sample of females without any cancer history. The WADLS is a comprehensive system linking population-based health and related data from several statutory datasets through probabilistic matching of routinely collected records from the same individual, with the proportion of valid links estimated through audits and validity studies to be>98.5% of matches [21]. The WA Cancer Registry (WACR), for which notification has been a statutory requirement since 1981 [22], was used to extract patients' basic demographic data, information on their tumor (date of diagnosis, anatomic site, histologic type). Tumors were classified by histologic type as described in the 3^rd^ edition of the *International Classification of Diseases for Oncology* [23], then grouped according to the Surveillance, Epidemiology, and End Results Program (SEER) AYA cancer diagnostic classification, a scheme developed to better define the major cancers that affect individuals between 15 and 39 years of age [24]. Tumors were further categorized by anatomic primary site as those arising in the (i) abdomen (ii) pelvis, or (iii) all other tumors. Exposure to cancer therapy was classified into one of five mutually exclusive treatment groups: surgery alone, chemotherapy alone, radiation therapy alone, chemotherapy plus radiation, and all other types and combinations of therapy. The Midwives Notification System (MNS) was used to obtain patients basic demographics and information on the pregnancy and delivery related to live births and stillbirths (20 weeks or more gestation or birth weight ≥400 g) in WA, 1982–2007. Information collected included maternal characteristics (age at delivery, marital status, ethnicity), pre-existing and new-onset health problems during the pregnancy, obstetric procedures and outcomes, and perinatal outcomes. The date of birth or pregnancy termination is registered with measurements of the newborn such as weight, length and vital status. The Hospital Morbidity Database System (HMDS), which lists principal and additional diagnoses and procedures, coded according to the *International Classification of Diseases* (ICD) editions 9 and 10, was used to extract data for all hospital admissions.

### Location and assignment of indices of socio-economic status

Socio-economic disadvantage was measured using Index of Relative Socio-economic Disadvantage (IRSD), which is based on census data of prevalence of low income, low educational attainment, high unemployment, rented dwellings, one parent families, and lacking fluency in English and other measures of social disadvantage [25]. The IRSD corresponded to the census collection district (CD) of the maternal residential address. Each CD contains approximately 200 dwellings.

### Selection of cases and comparison females

Cases were defined as females first diagnosed with histologically confirmed malignancy in WA while aged 15–39 years, in the period January 1, 1982 and Dec 31, 2007, and who had a subsequent delivery, either live or still birth, in WA on or before Dec 31, 2008. Only the first completed pregnancy (≥20 weeks) following cancer diagnosis was included. We created a frequency-matched comparison cohort using completed pregnancies of women with no registered history of cancer on the basis of maternal age (one year either way), delivery year (within 1 year), parity, and Aboriginal status. Any individual with a cancer diagnosis (primary or secondary) prior to their delivery was excluded from the comparison group.

### Adverse outcomes and exposure groups

Data on the obstetric and perinatal outcomes were obtained from the MNS based on diagnosis by the attending clinician/midwife. Adverse obstetric outcomes included the following: threatened abortion, threatened preterm labor, preterm delivery (gestation <37 weeks); preeclampsia (the onset of hypertension, i.e., systolic blood pressure> = 140 mm Hg and/or diastolic blood pressure> = 90 mm Hg from 20 weeks' gestation onwards accompanied by proteinuria); antepartum hemorrhage (defined as occurrence of placental abruption, placenta previa, or other excessive bleeding during labor and delivery); pre-labor rupture of membranes (PRoM: rupture of the membranes>12 h before onset of labor irrespective of gestation at the time of membrane rupture); gestational diabetes (diabetes first diagnosed during pregnancy, as confirmed by clinical investigations e.g., glucose tolerance test); other adverse pregnancy outcomes (e.g., intrauterine growth restriction (IUGR); intrauterine death (fetal death at ≥20 weeks of gestation), postpartum hemorrhage (> = 500 ml); Cesarean delivery. Adverse perinatal outcomes included: low birth weight (less than 2,500 g), low 1-min Apgar score (less <7), resuscitation (defined as the need for endotracheal intubation or external cardiac massage); admission to a special care unit; neonatal death (infant death during the 1st week of life); and congenital abnormalities identified prior to discharge from hospital.

### Statistical Analysis

Initial descriptive analysis followed by univariate analysis of study factors using Chi squared (*χ* ^2^) testing was performed. Stratified Mantel-Haenszel methods were applied to estimate relative risk (RR) with 95% confidence intervals (CI. The results were similar to those produced by log-binomial or Poisson models [26]. All models were adjusted for frequency-matched variables (parity, year of delivery, maternal age-group). Other variables used for adjustments were aboriginal status, residential remoteness, hospital insurance status, previous cesarean section, use of fertility treatment and maternal smoking during pregnancy. Gestational age was adjusted for in LBW. Sub-analyses were conducted, stratified by age at diagnosis, cancer SEER diagnostic groups, cancer anatomic site, calendar-period of diagnosis, and cancer treatment category. All analyses were conducted using SAS version 9.2 [27].

## Results

### Patient characteristics

A total of 1894 females were diagnosed with cancer in WA between 1982 and 2007 (Table 1). The majority of females were diagnosed with carcinoma (34%) or skin melanoma (25%). The proportion of AYA cancer patients identified with at least one subsequent pregnancy (>20 weeks) during the follow-up period was 24%. Table 2 compares the characteristics at index delivery of females diagnosed with AYA cancer with those of the group who had no cancer history. Distribution for maternal age, year of delivery, parity and Aboriginal status were similar between groups.

**Table 1:** Diagnostic characteristics and treatment details of AYAs diagnosis, Western Australia, 1982–2007.

	Cases n, %
***Total***	1894 (100%)
***Cancer diagnostic group***	
Leukemia	57 (3%)
Lymphoma	152 (8%)
CNS tumor	76 (4%)
Bone sarcoma	38 (2%)
Soft tissue sarcoma	95 (5%)
Germ cell tumor	208 (11%)
Melanoma	474 (25%)
Carcinoma	644 (34%)
Other	152 (8%)
***Cancer site***	
Abdomen	398 (21%)
Pelvis	170 (9%)
Other	1117 (59%)
***Age at cancer diagnosis (years)***	
15–19	739 (39%)
20–29	98 (52%)
30–39	170 (9%)
***Period of cancer diagnosis***	
1982–1988	322 (17%)
1989–1995	530 (28%)
1996–2001	587(31%)
2002–2007	455(24%)
***Type of cancer treatment***	
Surgery alone	644 (34%)
Chemotherapy alone	208 (11%)
Chemoradiation therapy	170 (9%)
Radiation therapy alone	170 (9%)
Other	701 (37%)

**Table 2:** Maternal characteristics of females diagnosed with AYA cancer and a comparison group who had no cancer history.

Characteristic	AYA cancer cohort, n (%)	Comparison cohort, n (%)
***Total***	1894 (100%)	4138 (100%)
***Maternal age at delivery (years)***		
15–19	193 (10%)	455 (11%)
20–29	841(44%)	1779 (43%)
30–34	550 (29%)	1242 (30%)
≥35	310 (16%)	662 (16%)
***Year of delivery***		
1982–1988	246 (13%)	497 (12%)
1989–1995	398 (21%)	828 (20%)
1996–2001	587 (31%)	1324 (32%)
2002–2007	663 (35%)	1489 (36%)
***Parity***		
Para 1	1023 (54%)	2193(53%)
Para2	417 (22%)	952 (23%)
Para 3 or more	454 (24%)	993 (24%)
***Indigenous status***	57 (3%)	83 (2%)
***Married/defacto status***	1686 (89%)	3931 (95%)
***Smoked during pregnancy***	284 (15%)	455 (11%)
***Genital herpes***	47 (3%)	86 (2%)
***Pre-existing diabetes***	114 (6%)	207 (5%)
***Asthma***	190 (10%)	335 (8%
***Previous cesarean section***	152 (8%)	455 (11%)
***Socioeconomic disadvantage***		
Low	587 (31%)	1366 (33%)
Mid	663 (35%)	1407 (34%)
High	644 (34%)	1366 (33%)
***Residential remoteness***	284 (15%)	952 (23%)
***Insurance status***		
Private	1155 (61%)	1821 (44%)
Public	739 (39%)	2317 (56%)

### Adverse maternal and neonatal outcomes

Comparative obstetric and perinatal complications after multivariate adjustment are reported in Table 3. Female survivors were nearly twice as likely to undergo fertility treatment compared with the non-cancer comparison group (adjusted relative risks, ARR 1.9, 95% Confidence Interval, CI 1.4–2.7). Females who had been diagnosed with AYA cancer had a higher risk of threatened abortion (2.1, 1.5–2.7), Pre-eclampsia (1.4, 1.1–1.9), gestational diabetes (2.7, 2.1–3.6), Cesarean delivery (2.6, 2.2–3.0), and were more likely to have a length of stay of longer than 5 days (3.0, 1.7–5.6) compared with the non-cancer group.

**Table 3:** Risk of maternal and perinatal adverse outcomes following births among females diagnosed with AYA cancer and comparison group.

	AYA cancer	Comparison	ARR (95% CI) ^a^
**Maternal complications**			
***Threatened abortion (<20 weeks)***	76(4%)	83 (2%)	2.09 (1.51–2.74)
***Threatened preterm labor (20–36 weeks)***	54 (3%)	91 (2%)	1.28 (0.88–1.88)
***Pre-eclampsia***	69 (4%)	111 (3%)	1.44 (1.13–1.87)
***Maternal anemia***	21(1%)	39 (1%)	1.31 (0.71–2.19)
***Gestational diabetes***	101 (5%)	83 (2%)	1.38 (1.09–2.98)
***Postpartum hemorrhage***	95 (5%)	199 (5%)	1.08 (0.82–1.56)
***Antepartum hemorrhage***	17 (1%)	41 (1%)	0.92 (0.59–1.78)
***PRoM***	99 (5%)	207 (5%)	0.99 (0.83–1.31)
***Failure to progress***	32 (2%)	47 (1%)	1.51 (0.97–2.37)
***Retained placenta***	57 (3%)	128 (3%)	0.98 (0.73–1.34)
***Cesarean delivery***	342 (18%)	288 (7%)	2.62 (2.22–3.04)
***Postpartum LOS>5 days***	227 (12%)	189 (5%)	3.01 (1.72–5.58)
***Use of fertility treatment***	57 (3%)	42 (1%)	1.94 (1.36–2.69)
**Perinatal complications**			
***Sex ratio (reference: male)***	948 (50%)	2029 (49%)	1.05 (0.98–1.10)
***Gestational age at birth***			
20–36 weeks	284 (15%)	412 (10%)	1.68 (1.21–2.08)
37–40 weeks	1458 (77%)	3310 (80%)	*Reference*
41–43 weeks	152 (8%)	416(10%)	1.04 (0.94–1.56)
**Birth weight**			
<2500 g	246 (13%)	331 (8%)	1.51 (1.23–2.12)
2500–4000 g	1439 (76%)	3435(83%)	*Reference*
>4000 g	208 (11%)	372 (9%)	1.33 (0.99–1.71)
***Intrauterine growth restriction***	119 (6%)	94 (2%)	1.21 (0.97–2.06)
***Low Apgar score at 1 min (<7)***	189 (10%)	124 (3%)	2.83 (2.28–3.56)
***Resuscitation***	164 (9%)	207 (5%)	1.66 (1.27–2.19)
***Admission to special/intensive care***	97(5%)	150 (4%)	1.44 (1.13–1.78)
***Intrauterine death***	38 (2%)	81 (2%)	1.07 (0.86–1.65)
***Neonatal death***	19 (1%)	41 (1%)	1.03 (0.54–1.71)
***Congenital abnormalities***	12 (1%	33 (1%)	0.78 (0.41–1.37)

Females in the cancer cohort were more likely to deliver infants at <37 weeks gestation (1.7, 1.2–2.1) or weighing <2500 grams (1.5, 1.2–2.1) relative to the comparison group. Infants born to females in the cancer cohort had an increased risk of resuscitation (1.7, 1.3–2.2), low Apgar score (<7) at 1 minute (2.8, 2.3–3.6), and admission to special care unit (1.4, 1.1–1.8). The male: female ratios of offspring were similar in the cancer cohort and comparison group. Intrauterine and neonatal deaths and congenital abnormalities occurred in similar proportions in both cohorts.

### Outcomes by diagnostic and treatment categories

Tables 4 shows the risks (ARR) of adverse obstetric and neonatal complications respectively, among females with previous cancer compared with those without, according to AYA cancer diagnostic characteristics and treatment details. Risk of all four obstetric outcomes was highest for older women (30–39 years) and in the 2002–2007 calendar period of diagnosis. All adverse outcomes were also most likely after diagnosis of CNS tumor (except cesarean section) and carcinomas. Risk of cesarean section was high after bone carcinoma and the risk of maternal diabetes was high following diagnosis of almost all of the cancer types. All adverse outcomes were slightly more likely following a diagnosis of a cancer in the pelvis. Risk of threatened abortion and pre-eclampsia were more likely following radiation therapy alone rather than other treatment types, but maternal diabetes was more likely after chemoradiation and cesarean section was most likely after chemotherapy alone.

**Table 4:** Selected adverse obstetric and perinatal outcomes according to AYA cancer characteristics and treatment details, Adjusted Relative Risk versus non-cancer comparison group (95% CI)^a^.

	Adjusted relative risk (95% CI) versus non-cancer comparison group						
Cancer characteristic	Threatened abortion	Gestational diabetes	Cesarean delivery	Preterm delivery	LBW	Low Apgar score (<7)	Resuscitation
***Age at diagnosis (years)***							
15–19	0.76 (0.42–1.68)	1.12 (0.50–3.96)	0.66 (0.47–1.88)	1.32 (0.97–1.94)	1.34 (0.97–1.81)	1.34 (0.81–2.43)	1.13 (0.68–1.72)
20–29	1.15 (0.58–3.98)	1.64 (0.98–2.85)	1.22 (0.97–3.32)	1.66 (0.99–2.68)	1.75 (1.17–2.64)	2.24 (1.56–3.65)	1.35 (0.72–2.81)
30–39	1.58 (1.09–3.43)	3.11 (1.10–7.98)	3.16 (1.01–10.0)	1.72 (1.29–1.86)	1.63 (1.26–2.82)	1.81 (1.01–2.68)	1.68 (1.18–2.35)
***Diagnosis period***							
1982–1988	1.06 (0.74–1.51)	0.88 (0.44–6.11)	1.12 (0.84–2.52)	1.21 (0.86–1.70)	0.88 (0.44–2.32)	1.48 (0.75–1.42)	0.98 (0.67–2.15)
1989–1995	0.64 (0.27–1.46)	0.92 (0.28–4.32)	1.38 (0.86–2.03)	1.42 (0.96–1.90)	1.11 (0.63–4.19)	1.31 (0.52–2.94)	0.92 (0.29–2.90)
1996–2001	1.29 (0.98–1.82)	1.28 (1.02–2.66)	2.16 (0.97–3.97)	1.32 (0.97–2.97)	1.16 (0.98–2.45)	2.44 (1.18–4.59)	1.16 (0.98–1.67)
2002–2007	2.19 (1.32–3.13)	1.92 (0.82–3.98)	2.32 (1.08–4.16)	1.88 (1.12–2.72)	1.43 (1.05–2.06)	1.56 (0.93–2.67)	1.34 (1.06–2.70)
***Diagnostic type***							
Leukemia	2.18 (0.90–5.70)	1.23 (0.19–9.44)	2.01 (1.26–12.4)	1.71 (1.18–2.41)	1.79 (1.43–2.56)	1.51 (0.77–3.24)	0.83 (0.43–2.47)
Lymphoma	1.08 (0.44–2.77)	1.52 (0.78–2.83)	1.45 (0.95–2.18)	0.94 (0.38–2.34)	0.87 (0.26–2.36)	1.04 (0.54–1.87)	1.48 (0.42–5.83)
CNS tumor	2.14 (1.13–3.76)	2.32 (1.29–3.98)	0.99 (0.77–1.45)	1.81 (1.37–2.54)	0.97 (0.66–1.51)	0.92(0.67–1.34)	0.92 (0.45–1.84)
Bone sarcoma	1.25 (0.84–1.73)	2.14 (1.12–6.08)	2.14 (1.34–3.85)	1.18 (0.56–2.00)	1.16 (0.86–1.87)	1.43 (0.76–1.57)	1.15 (0.86–1.72)
Soft tissue sarcoma	0.81 (0.09–4.93)	2.73 (0.41–18.8)	1.38 (0.45–2.31)	0.87 (0.38–2.44)	1.73 (0.82–3.64)	1.09 (0.94–1.93)	0.96 (0.42–6.18)
Germ cell tumor	0.96 (0.32–6.18)	2.91 (0.52–18.8)	0.92 (0.54–6.08)	0.91 (0.28–2.94)	1.38 (1.00–1.82)	2.64 (1.14–4.88)	1.07 (0.81–1.84)
Melanoma	0.71 (0.46–4.93)	2.01 (0.34–12.8)	1.57 (0.94–2.44)	1.08 (0.73–1.91)	1.05 (0.55–1.97)	1.77 (0.32–4.91)	0.86 (0.43–3.12)
Carcinoma	2.41 (1.35–6.47)	2.45 (1.28–4.04)	2.34 (1.17–7.68)	2.39 (1.72–3.85)	2.13 (1.24–3.59)	1.41 (1.04–2.95)	1.67 (1.18–3.09)
***Anatomical site***							
Abdomen	1.73 (1.12–2.52)	1.32 (1.09–2.57)	1.38 (1.14–2.32)	1.56 (1.23–2.83)	1.41 (0.99–2.24)	2.16 (0.98–3.94)	1.63 (1.22–2.34)
Pelvis	2.02 (1.08–3.18)	2.61 (1.83–3.97)	2.14 (1.34–3.85)	1.76 (1.34–2.97)	1.88 (1.24–2.87)	2.21 (1.09–3.40)	1.18 (0.72–2.09)
***Treatment type***							
Chemotherapy alone	1.48 (0.87–2.34)	1.25 (0.31–4.99)	1.78 (1.27–2.49)	1.28 (0.99–2.14)	1.25 (0.57–2.98)	0.98 (0.34–5.64)	1.84 (1.19–4.54)
Radiation alone	1.98 (1.38–2.59)	0.80 (0.25–2.56)	1.35 (1.11–2.80)	1.78 (1.53–3.74)	1.82 (1.26–2.59)	2.14 (1.13–3.96)	1.63 (0.94–2.72)
Surgery alone	1.02 (0.52–1.85)	1.34 (0.18–9.84)	0.84 (0.58–1.64)	0.94 (0.32–2.94)	0.98 (0.23–2.44)	1.08 (0.83–1.72)	1.17 (0.96–1.62)
Chemoradiation	1.08 (0.54–1.87)	2.52 (1.12–5.09)	1.45 (0.96–2.09)	1.05 (0.43–2.88)	1.52 (1.01–2.43)	1.78 (1.11–3.04)	1.04 (0.55–2.29)
Other/unknown	1.30 (0.97–1.76)	0.90 (0.29–2.81)	1.07 (0.78–1.88)	1.21 (0.43–2.78)	1.05 (0.89–2.12)	1.11 (0.74–1.61)	2.82 (0.37–12.8)

a The final models were adjusted for: Aboriginal status, previous cesarean section, maternal smoking, use of fertility treatment, residential remoteness, hospital status.

The risks of all adverse perinatal outcomes tend to risk with increasing age of diagnosis. With the exception of low Apgar score, adverse perinatal outcomes were mostly likely to occur in the most study periods. All adverse outcomes were more likely in the offspring of females previously diagnosed with carcinomas. The risk of preterm delivery and LBW, resuscitation were highest in the offspring of survivors of leukemia. The risk of preterm delivery, LBW and resuscitation were highest among offspring of women with malignancies arising in the pelvis. The offspring of women exposed to radiation had a high risk of preterm delivery, LBW and low Apgar score whereas resuscitation was most likely in women who were exposed to chemotherapy.

No significant increases in risks for antepartum hemorrhage, postpartum hemorrhage, IUGR, PRoM, failure to progress, retained placenta, intrauterine death or neonatal death were observed across cancer diagnostic and treatment categories (data not shown).

## Discussion

### Main findings

In this large study of pregnancy outcomes following AYA cancer, female survivors had a moderately increased risk of obstetric complications such as threatened abortion, cesarean delivery, pre-eclampsia and gestational diabetes, compared to females with no history of cancer. The offspring of female survivors had a higher risk of preterm birth and LBW, and measures of neonatal distress (low Apgar score at one minute, need for resuscitation, and admission to a special care unit) compared to the offspring of females from the non-cancer cohort.

Subgroup analyses according to AYA cancer diagnostic characteristics and treatment details found that threatened miscarriage was higher in survivors of AYA cancer diagnosed at an older age, those with a history of CNS tumors and carcinomas, abdominopelvic site tumors as well as females who had been treated with radiotherapy. Generally speaking, the most important antecedent to miscarriage is chromosomal abnormality [28]. Other factors which influence the risk of miscarriage include older maternal age, congenital uterine abnormalities, autoimmune factors, thrombophilic disorders, maternal endocrine abnormalities (e.g., poorly controlled diabetes and polycystic ovarian syndrome) [29]. Although it is plausible that conditions such as autoimmune or metabolic disorders may occur secondarily to cancer or its treatment, the specific reasons for threatened miscarriage in this group of females is still unclear. However, the increased risk of threatened miscarriage among survivors of pelvic tumors and those who had been treated with radiation raises the possibility that this outcome is an adverse effect of prior uterine irradiation [30]. Overall, there was no strong indication that threatened abortion varied by cancer type except among females diagnosed with CNS tumors and carcinoma. The CNS finding suggests that radiation to the brain may increase the likelihood of a miscarriage, possibly through impairment of the hypothalamic-pituitary-ovarian-axis [31], [32].

We found that gestational diabetes featured more frequently among survivors of CNS tumours, bone sarcoma and carcinomas and patients with tumours arising in the abdominal pelvic region. Our study also found maternal diabetes was more common in females exposed to chemoradiation. Possible associations between maternal diabetes and cancer subgroup are largely understudied. A few studies have reported that childhood cancer survivors whose treatment included cranial [33], [34] and total body irradiation [35] were at risk of diabetes mellitus. The sequelae from cancer therapies, such as chemotherapy and radiotherapy, may potentially compromise health in several ways that could lead to decreased immune functioning, cardiotoxic effects, and weight gain [32], which may in turn contribute to secondary health problems such as cardiovascular disease and diabetes.

Cesareans were more common in females diagnosed with cancer compared with those with no history of cancer and in particular common in those females exposed to chemotherapy or radiotherapy, and females diagnosed with leukemia, bone cancer and primary cancers in the abdomen or pelvis. The combination of psychological and obstetric considerations has probably led to the high frequency of cesarean delivery in females diagnosed with cancer. Possible clinician- and patient-dependent reasons for an increased rate of planned cesarean sections, including concerns over medical malpractice, fear of birth trauma and the potential risk to the child due to difficult vaginal delivery. However, this does not necessarily explain the specific difference identified within the observed results and therefore requires further in-depth investigations.

The excess risk of low birth weight and preterm delivery among females treated by radiation therapy is likely related to the radiation dose to the uterus irrespective of cancer type [36], but it is difficult to distinguish between treatment effects and cancer type. Survivors of certain cancer sub-types (e.g., lymphoma, soft tissue sarcoma and carcinomas) had an increased risk of preterm delivery and offspring with LBW. Previous investigators have hypothesized that radiation-induced damage to abdominopelvic tissue, including vasculature, could interfere with fetal growth by physical constraint of uterine volume or by restricting vascular support to the pregnancy, leading to low birth weight or small for gestational age [11], [19], [37]. Also, uterine fibrosis might affect cervical competence or placentation which are both associated to preterm delivery [32], [38]–[42].

Overall the offspring of female survivors of cancer were more likely to experience fetal distress. The Apgar score, which is assigned to virtually every newborn, evaluates the clinical state of the newborns based on five physical signs (heart rate, respiratory effort, reflex irritability, muscle tone and color) present shortly after birth [43]. A higher risk of low Apgar score was observed among offspring of females who had a history of germ cell tumors and carcinoma and those exposed to radiation therapy or chemoradiation. A low Apgar score is a marker of a suboptimal fetal environment. The mechanism underlying this observation is, however, unclear.

It is worth noting that, the gender ratio of the offspring of the females in our study was not significantly different from that of the comparison group. This is reassuring because it suggests that there is no deficit of male infants among the offspring of the female survivors, a finding that, were it present, would suggest transmission of lethal X-linked mutations [44].

The finding that more female cancer patients used fertility treatment than the non-cancer comparison group was not surprising. We were unable to further investigate specific associations between cancer diagnosis and fertility due to lack of detail information regarding treatment exposure. However, a number of past studies have found that certain chemotherapy treatment regimens [45], especially those including high-dose alkylating agents, can lead to infertility. Pelvic irradiation can also adversely affect ovarian function, whereas cranial radiation can impair the hypothalamic pituitary function and cause hypogonadism through gonadotropin-releasing hormone (GnRH) deficiency [46] and total-body irradiation in hematologic malignancies affects uterine volume [47]. Due to these potentially serious long-term fertility consequences, female patients should be informed of available methods of fertility pereservations before the initiation of cancer-directed therapy. Currently, there are several possibilities to preserve future fertility, including in vitro fertilization (IVF) and embryo cryopreservation, ovarian tissue cryopreservation, unfertilized ova cryopreservation, and the administration of a GnRH agonist [48]–[51].

### Strengths and Limitations

This retrospective study used routinely collected statutory data for the entire population of Western Australia, which provided a large sample size and minimized incomplete case ascertainment and loss to follow-up between cancer diagnosis and pregnancy. The MNS provided us with comprehensive data about gestational, delivery, and infant outcomes of completed pregnancies, but excluded pregnancies resulting in completed miscarriage (<20 weeks). Information from the registries included important confounders and well-established risk factors for adverse outcomes, such as smoking. However, we did not have detailed information about cancer therapies, such as radiation dose and field location or specific chemotherapeutic agents. For example, lack of comprehensive treatment information precluded investigating the risk of adverse pregnancy outcomes by dose of radiation received to the reproductive organs; hence, residual confounding by radiation exposure could have distorted the magnitude of the measured associations. Furthermore, survivors included in this investigation were treated between 1982 and 2007 and hence, less is known about the potential adverse effects of more recent therapies which are believed to be much more aggressive than earlier therapies. Survivors treated more recently are still relatively young and the number of offspring born to these survivors will be relatively small. There are inevitable caveats on interpretation of findings from subgroup analyses, particularly as the routinely collected data in this study lacked certain important clinical details, such that causal inferences are necessarily speculative. Further, we lacked information about the patient's childbearing intent, the number and timing of attempts of post-treatment parenthood or whether assisted reproductive technology was actually used because of patient or partners subfertility. Therefore our findings are primarily relevant to females who have attempted parenthood and were able to become pregnant. It is also worth noting that the recording of a number of maternal conditions such as diabetes may be less sensitive, albeit highly specific and that the differential monitoring of females with a cancer history could have resulted in the increased identification of some prenatal conditions.

### Past studies

There are number of previous reports of pregnancy outcomes in long-term survivors of diverse types of pediatric cancer [10]–[20]. However, only a few published studies have specifically focused on patients of childbearing age at the time of cancer diagnosis [52]–[56]. Like our study, these studies reported significantly elevated risks of preterm delivery and LBW, ranging from 1.3–3.1 [53], [56], [57] and 2.0–3.7 [56], [57], respectively. Two studies reported additionally that infants born to female AYA cancer survivors were also at higher risk of perinatal death [1.9–2.3] compared to offspring of females with no prior cancer diagnosis [56], [57]. After adjustment for prematurity, another study found that the risk of early death or stillbirth was not increased [54]. One study found that cancer survivors had higher rates of postpartum hemorrhage (OR 1.56) and operative or assisted delivery (OR 1.33) compared with females without a history of cancer [55]. The principal limitations in these recent studies investigating outcomes in AYA females arises from the fact that some past studies using hospital based data from single institutions, which may have hindered accurate or valid quantification of risks due to small sample size [57]; another study focused exclusively on a few neonatal outcomes (preterm delivery, LBW) and did not quantify maternal-related complications [53], [56]. Although one study extended the age range of cancer survivors by including patients aged 0 to 43 years, they did not separately report diagnostic age-specific risk estimates for AYAs [55]. Our study presents a detailed assessment of maternal outcomes in female survivors of adolescent and young adult cancers.

## Conclusions

Our data indicate that AYA cancer survivors who are have an excess risk of threatened abortions, cesarean delivery, preeclampsia, diabetes and their offspring have a moderately elevated risk of preterm birth and LBW. Our findings also suggest an adverse association between older age at diagnosis, certain cancer diagnoses and therapies, namely radiation therapy and cancer in the abdominal-pelvic region. Although their infants may be more likely to be preterm or of low birth weight, we observed no increases in congenital malformations, or neonatal death and no altered male to female sex ratio that might indicate increased germ cell mutagenicity. Overall, our results indicate a need for close surveillance of female survivors of AYA cancers. Understanding the effects of cancer on future childbearing may assist in the strategic targeting of resources to give these females the best care and access to treatment.

## Ethics Statement

This study protocol, including the use of de-identified, administrative health data without patient consent, was approved by the Human Research Ethics Committee of The University of Western Australia and the Department of Health Western Australia Human Research Ethics Committee (RA/4/1/2229). This study was performed in accordance with the Declaration of Helsinki.
